# Phosphate-solubilizing bacteria-assisted phytoremediation of metalliferous soils: a review

**DOI:** 10.1007/s13205-014-0206-0

**Published:** 2014-03-12

**Authors:** Munees Ahemad

**Affiliations:** Department of Agricultural Microbiology, Faculty of Agricultural Sciences, Aligarh Muslim University, Aligarh, 202002 UP India

**Keywords:** Bioremediation, Heavy metals, Hyperaccumulator plants, Phosphate-solubilizing bacteria, Phytoremediation, Rhizobacteria

## Abstract

Heavy metal pollution of soils is of great concern. The presence of the toxic metal species above critical concentration not only harmfully affects human health but also the environment. Among existing strategies to remediate metal contaminates in soils, phytoremediation approach using metal accumulating plants is much convincing in terms of metal removal efficiency, but it has many limitations because of slow plant growth and decreased biomass owing to metal-induced stress. In addition, constrain of metal bioavailability in soils is the prime factor to restrict its applicability. Phytoremediation of metals in association with phosphate-solubilizing bacteria (PSB) considerably overcomes the practical drawbacks imposed by metal stress on plants. This review is an effort to describe mechanism of PSB in supporting and intensifying phytoremediation of heavy metals in soils and to address the developmental status of the current trend in application of PSB in this context.

## Introduction

Soil is one of the most important natural resource on which lives of all plants, animals and microorganisms directly or indirectly dependent. In soils, different microorganisms thrive on abundantly present nutrients therein and through various interactions play a pivotal role in cycling of nutrients and pedogenesis (Ahemad and Khan 2013). Alteration or disturbance in soil ecosystem by added pollutants leads to substantial changes in functional activities of these important soil microorganisms (Swain and Abhijita 2013). Among pollutants, enormous amounts of toxic heavy metals such as chromium, cadmium, copper, zinc, mercury and lead contaminate soils through various geogenic, anthropogenic and technogenic activities (Ahemad 2012; Liu et al. 2013; Waterlot et al. 2013; Chodak et al. 2013). Due to non-biodegradable nature, metals in soils persist longer and pose a risk to human health through food chain because of their carcinogenicity, mutagenicity and teratogenicity (Ahemad and Malik 2011; Ali et al. 2013; Ahemad and Kibret 2013a). In addition, metals exceeding threshold limit affect microbial diversity and soil fertility (Huang et al. 2009). Thus, remediation of such metal-stressed soils is of paramount significance as they are rendered inappropriate for agricultural application.

Many physicochemical technologies are already in practice to clean up the metal-contaminated soils (Hashim et al. 2011). However, these conventional technologies are generally too costly to be applied to decontaminate the metal-polluted sites. Moreover, they generally adversely affect the texture and organic components, which are important to sustain the fertility of soils (Rajkumar et al. 2010). In view of sustainability issues and environmental ethics, bioremediation, the exploitation of biological processes for the cleanup of contaminated sites, is a promising, benign and ecologically sound alternative to chemical technologies (Hashim et al. 2011; Gillespie and Philp 2013). Among different bioremediation approaches, phytoremediation (utilizing metal accumulating plants to detoxify and extract contaminants in polluted soils) is gaining wide acceptance due to being cheap and environmentally safe but the major drawback of this technique is that it is time-consuming and high levels of metals decease the remediating efficiency of plants (Ali et al. 2013). Interestingly, interactions between plant and metal resistant bacteria have shown better remediation of heavy metals, and this synergism not only expedites the remediation process by ameliorating phytostabilization (reduction in metal toxicity through metal immobilization) and phytoextraction (metal accumulation as a result of metal mobilization) of metal species but also accelerate the plant growth and development (Khan et al. 2009).

Since last decades, several phosphate-solubilizing bacteria (PSB) exhibiting both heavy metal detoxifying traits and plant growth promoting activities have been explored and have been implicated in phytoremediation of metalliferous soils (He et al. 2010; Misra et al. 2012; Oves et al. 2013; Ahemad and Kibret 2013b). This review is an effort to emphasize how the beneficial association between plants and PSB can be used to remediate the metal-stressed soils efficiently. In this review, mechanism of PSB mediation in supporting and intensifying phytoremediation process is discussed in detail.

## Phytoremediation: an overview

Currently, several physicochemical and biological techniques are in practice to remediate the metal-contaminated soils. Of them, remediation processes based upon the physicochemical parameters are very costly and also affect the soil properties, biodiversity and fertility. Different remediation technologies have been compared in Table 1 in terms of cost. It is obvious from the enlisted remediation approaches that phytoextraction (phytoremediation type) is one of the most cost effective method to remediate the metal-polluted soils (Padmavathiamma and Li 2007). Phytoremediation occurs only at marginal cost, which is due to harvesting and field management, e.g., weed control. In addition, the resulting biomass of phytoremediating plants can be used for heat and energy production in specialized facilities (Peuke and Rennenberg 2005). Unlike physicochemical processes, phytormediation is an eco-friendly and comprehensive strategy having no side effects on soil texture and health (Suresh and Ravishankar 2004).

**Table 1:** Cost of different remediation technologies

Process	Cost (US$/ton)	Other factors
Vitrification	75–425	Long-term monitoring
Land filling	100–500	Transport/excavation/monitoring
Chemical treatment	100–500	Recycling of contaminants
Electrokinetics	20–200	Monitoring
Phytoextraction	5–40	Disposal of phytomass

*Source*: Glass (1999)

In phytoextraction, metals are accumulated in plant biomass from moderately contaminated soils. On the other hand, phytostabilization is a long-term, in situ approach applicable in polymetallic soils wherein concentration and the area of metal contaminant are so extensive that phytoextraction cannot work; therefore, metals are not allowed to enter plants but are captured in situ through biosorption, precipitation or reducing toxicity (de-Bashan et al. 2012). Plants selected for phytostabilization must be metal-tolerant (metallophytes) and should not accumulate metals into root tissues. Despite having mechanisms to evade metal translocation in shoot tissues, considerable amount of metals may be found in the shoot parts. Metal accumulation in plants can measured in terms of (1) bioconcentration factor (BF)/accumulation factor (AF) which is the ratio of metal concentration in the shoot tissue to the soils and (2) translocation factor (TF) which is the ratio of metals in shoot to those in root. For better phytostabilization, these values should preferably be ≪1 while the values must be ≫1 in addition to higher plant biomass for an ideal phytoextracting plants (Peuke and Rennenberg 2005; Mendez and Maier 2008).

## Phosphate-solubilizing bacteria

After nitrogen, phosphorus (P) is the second essential macronutrient for plant growth and development. Generally, substantial amount of phosphorus (P) occurs in soil ranging from 400 to 1,200 mg/kg of soil, either in mineral forms, e.g., apatite, hydroxyapatite and oxyapatite, or organic forms such as, inositol phosphate (soil phytate), phosphomonoesters, phosphodiesters and phosphotriesters (Ahemad et al. 2009). However, the concentration of soluble forms of P in soil is usually ~1 mg/kg or less (Goldstein 1994). In addition, it has a very limited bioavailability to growing plants due to high reactivity of phosphate ions in soils. To circumvent this deficiency, phosphatic fertilizers are applied in soils. But most of the applied P in the forms of fertilizers is precipitated; consequently, a very small fraction is available for absorption by plants. As an eco-friendly and economical alternative to provide substantial amount of soluble P to plants for growth promotion is the exploitation of P solubilization and mineralization traits of PSB.

**Fig. 1:**
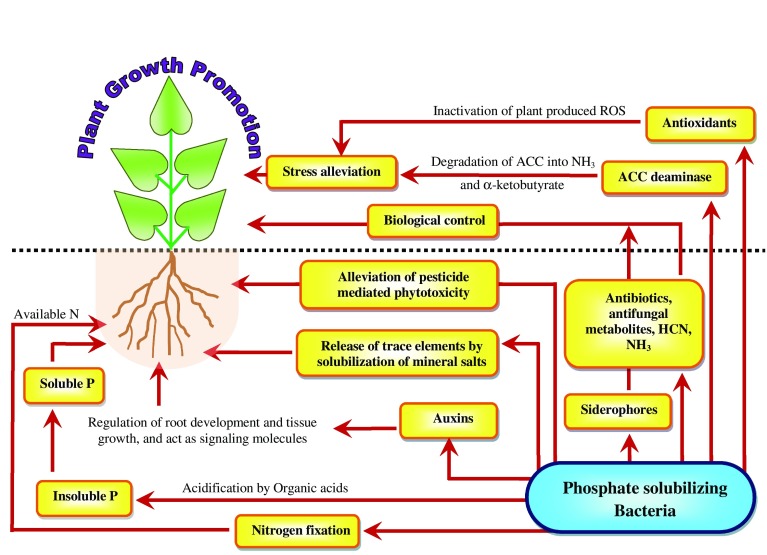
Mechanisms of phosphate-solubilizing bacteria-mediated plant growth promotion. *ROS* reactive oxygen species, *ACC* 1-aminocyclopropane-1-carboxylate, *NH*_*3*_ ammonia, *HCN* hydrogen cyanate, *IAA* indole-3-acetic acid, *P* phosphate

Additionally, PSB not only protect plants from phytopathogens through the production of antibiotics, HCN, phenazines and antifungal metabolites, etc. (Upadhayay and Srivastava 2012; Singh et al. 2013), but also promote plant growth through N_2_ fixation (He et al. 2010), siderophore production (Ahemad and Khan 2012a, b), phytohormone secretion (Misra et al. 2012; Oves et al. 2013) and lowering ethylene levels (Jiang et al. 2008; Kumar et al. 2009) (Fig. 1).

## Mechanisms of PSB-assisted metal phytoremediation

**Fig. 2:**
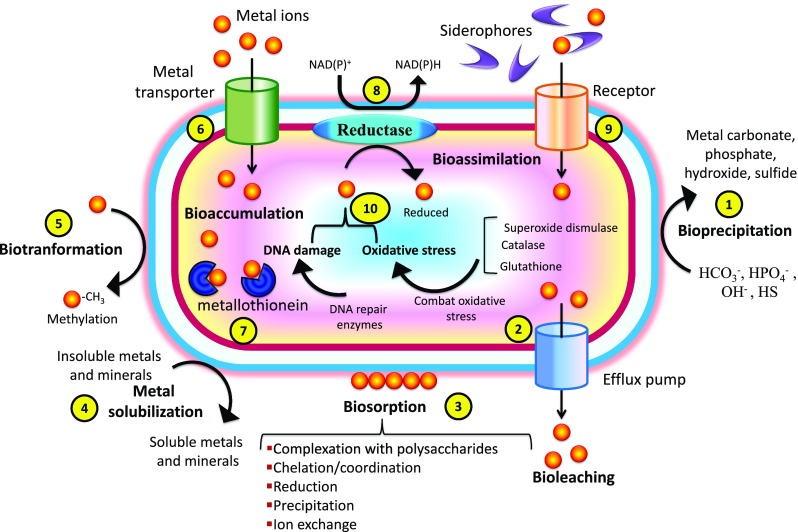
Various bacterial interactions with heavy metals in metal-polluted soils: *1* precipitation/crystallization of metals occurs due to bacteria-mediated reactions or as a result of the production of specific metabolites. *2* Plasmid-DNA-encoded efflux transporters (e.g., ATPase pumps or chemiosmotic ion/proton pumps) expel the accumulated metals outside the cell. *3* Metals bind to the anionic functional groups (e.g., sulfhydryl, carboxyl, hydroxyl, sulfonate, amine and amide groups) of extracellular materials present on cell surfaces. *4* Organic acids secreted by bacteria solubilize the insoluble metal minerals. *5* Some bacteria utilize methylation as an alternative for metal resistance/detoxification mechanism, which involves the transfer of methyl groups to metals and metalloids. *6* Metals enter the bacterial cell by chromosomal DNA-encoded metal transporters either through ATP hydrolysis or as a result of chemiosmotic gradient across the cytoplasmic membrane. *7* Bacterial cell also accumulate substantial concentration of metals by the synthesis of low molecular mass cysteine-rich metal-binding proteins, metallothioneins having high affinities for several metals. *8* Membrane-embedded metal reductases, generally encoded by chromosomal DNA, reduce metals in the presence of electron donors. *9* Siderophore secretion decreases metal bioavailability by binding metal ions having chemistry similar to iron. *10* Superoxide dismutase, catalase and glutathione are activated to combat oxidative stress produced by the reactive oxygen species (ROS), and DNA repair system is activated to repair the DNA damaged due to various metal interactions within cell

**Fig. 3:**
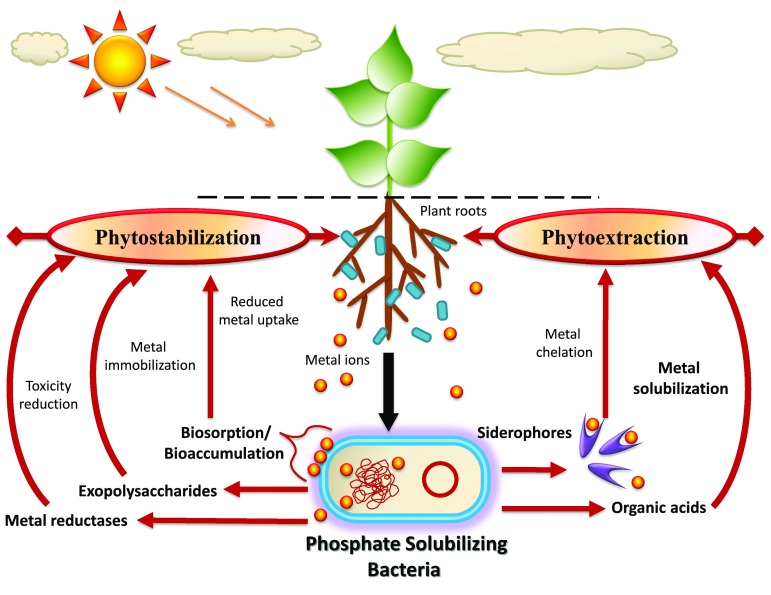
Schematic portrayal of the role of metal resistant phosphate-solubilizing bacteria in alleviation of heavy metal toxicity, phytoextraction and phytostabilization

Although decontamination of metal-polluted soils using plants (phytoextraction/phytostabilization) has shown encouraging results, this approach has limitations in case of the polluted sites wherein metal concentration is extremely elevated (Gamalero and Glick 2012). Under high metal stress, their physiological activities are hampered; growth and development are severely impeded; and resistance mechanisms are weakened, and in turn, they become prone to phyto-pathogen attacks (Ma et al. 2011c). Further, their metal phytoremediating efficiency is depressingly affected, and the process of metal decontamination is proportionally impeded depending upon several factors (Martin and Ruby 2004). Intended to overcome the noxious level of metals that significantly decline the plant growth, PSB with multiple plant growth promoting traits (Table 2; Fig. 1) and concurrent metal detoxifying potentials (Fig. 2) may increase the phytoremediation competence of plants by promoting their growth and health even under hazardous levels of different metals. As adjuncts with plants, PSB remediate metal-contaminated soils largely through facilitating either phytostabilization (decreasing metal toxicity by transforming metal species into immobile forms) or phytoextraction (metal mobilization and accumulation in plant tissues) (Fig. 3). Various plant growth promoting traits of PSB, such as organic acid production, secretion of siderophores, IAA production and ACC deaminase activity, contribute in enhancing the phytoremediation capability of plants.

**Table 2:** Plant growth promoting substances released by phosphate-solubilizing bacteria

PGPR	Plant growth promoting traits	References
Pseudomonas aeruginosa strain OSG41	IAA, siderophores	Oves et al. (2013)
*Pseudomonas* sp.	IAA, HCN	Singh et al. (2013)
*Acinetobacter haemolyticus* RP19	IAA	Misra et al. (2012)
*Pseudomonas putida*	IAA, siderophores, HCN, ammonia	Ahemad and Khan (2011c, 2012b, c)
*Pseudomonasfluorescens* strain Psd	IAA, siderophores, HCN, antibiotics, biocontrol activity	Upadhayay and Srivastava (2012)
*Bacillus thuringiensis*	IAA	Sandip et al. (2011)
*Pseudomonas aeruginosa*	IAA, siderophores, HCN, ammonia	Ahemad and Khan (2010c, 2011a, e, 2012d)
*Pseudomonas* sp. TLC 6-6.5-4	IAA, siderophore	Li and Ramakrishna (2011)
*Bacillus* sp.	IAA, HCN	Karuppiah and Rajaram (2011)
*Klebsiella* sp.	IAA, siderophores, HCN, ammonia	Ahemad and Khan (2011b, d, 2012a)
*Enterobacter asburiae*	IAA, siderophores, HCN, ammonia	Ahemad and Khan (2010a, b)
*Bacillus* species PSB10	IAA, siderophores, HCN, ammonia	Wani and Khan (2010)
*Arthrobacter* sp. MT16, *Microbacterium* sp. JYC17, *Pseudomonaschlororaphis* SZY6, *Azotobactervinelandii* GZC24, *Microbacteriumlactium* YJ7	ACC deaminase, IAA, siderophore	He et al. (2010)
*Pseudomonas* sp.	IAA, siderophore, HCN, biocontrol potentials	Tank and Saraf (2009)
*Enterobacteraerogenes* NBRI K24, *Rahnellaaquatilis* NBRI K3	ACC deaminase, IAA, siderophore	Kumar et al. (2009)
*Enterobacter* sp.	ACC deaminase, IAA, siderophore	Kumar et al. (2008)
*Burkholderia*	ACC deaminase, IAA, siderophore	Jiang et al. (2008)
*Pseudomonas aeruginosa*	ACC deaminase, IAA, siderophore	Ganesan (2008)

*ACC* 1-aminocyclopropane-1-carboxylate, *HCN* hydrogen cyanate, *IAA* indole-3-acetic acid

## Organic acids

In most of the metalliferous soils, metals are strongly adhered to soil particles; therefore, they are not easily available for uptake by phytoextracting plants (Gamalero and Glick 2012). In this context, PSB are very promising agents since they solubilize the insoluble and biologically unavailable metals such as Ni (Becerra-Castro et al. 2011), Cu (Li and Ramakrishna 2011) and Zn (He et al. 2013) by secreting low molecular weight organic acids; thus, they facilitate metal bioavailability for plant uptake (Becerra-Castro et al. 2011). A number of organic acids such as lactic, citric, 2-ketogluconic, malic, glycolic, oxalic, malonic, tartaric, valeric, piscidic, succinic and formic have been identified, which have chelating properties (Panhwar et al. 2013). Moreover, metal bioavailability in metal-stressed soils can be further increased by inoculating biosurfactant producing PSB as the bacterial biosurfactants aid in metal release from soil particles (Gamalero and Glick 2012; Singh and Cameotra 2013).

## Siderophores

Generally, iron occurs mainly as Fe^3+^ and forms insoluble hydroxides and oxyhydroxides, thus is not easily available to both plants and microorganisms (Ahemad and Kibret 2013b). Under iron-limiting conditions to acquire iron, bacteria secret low molecular weight siderophores, which are iron chelators with exceptionally strong affinity for ferric iron (Fe^3+^) (Schalk et al. 2011). Despite their preferential affinity for Fe^3+^, they can also chelate several other metals such as, magnesium, manganese, chromium (III), gallium (III), cadmium, zinc, copper, nickel, arsenic and lead, and radionuclides, including plutonium (IV) with variable affinities (Nair et al. 2007; Rajkumar et al. 2010; Schalk et al. 2011). Supply of iron to growing plants under heavy metal pollution becomes more important as bacterial siderophores help to minimize the stress imposed by metal contaminants (Gamalero and Glick 2012). For instance, siderophore overproducing mutant NBRI K28 SD1 of phosphate-solubilizing bacterial strain *Enterobacter* sp. NBRI K28 not only increased plant biomass but also enhanced phytoextraction of Ni, Zn and Cr by *Brassicajuncea* (Indian mustard) (Kumar et al. 2008).

## Indole acetic acid

Phytohormone, indole-3-acetic acid (IAA) whose biosynthesis requires l-tryptophan as a precursor, is the most important auxin, which regulates several morphological and physiological functions in plants (Glick 2012). Although it has been implicated in stimulation of root growth, alleviation of salt stress, plant-pathogen interactions, legume-rhizobia interactions and eliciting induced systemic resistance against various diseases, it primarily is involved in stimulating the proliferation of lateral roots in plants, thereby root surface area is increased and they absorb more water and soil minerals (Egamberdieva 2009; Lugtenberg and Kamilova 2009, Ahemad and Kibret 2013b). Many phosphate-solubilizing bacterial genera (He et al. 2010; Ahemad and Khan 2011a, 2012b; Misra et al. 2012; Oves et al. 2013) in soils have been reported to secret IAA that is absorbed by plant roots to increase the endogenous pool of plant IAA (Glick et al. 2007). However, effects of variable IAA concentrations vary among different plant species. Moreover, optimum concentration of bacterial IAA has stimulatory effect, while high concentration (supra-optimal) of those is inhibitory to root growth (Glick 2012).

Generally, bacterial IAA facilitates adaptation of host plants in metal-contaminated sites through triggering physiological changes in plant cell metabolism under metal stress so that the growing plants can withstand high concentrations of heavy metals (Glick 2010). However, Hao et al. (2012) determined that bacterial IAA had a larger impact on the growth of host plants under metal stress rather than bacterial metal resistance through transposon mutagenesis in phosphate-solubilizing *Agrobacterium tumefaciens* CCNWGS0286.

## 1-Aminocyclopropane-1-carboxylate (ACC) deaminase

Another phytohormone, ethylene, modulates many important physiological activities of growing plants including root growth and development. Under both biotic (e.g., phyto-pathogen attacks) and abiotic (e.g., heavy metals, drought, flooding and salinity) stresses, plant produces ethylene up to the level that is inhibitory to root growth (Khalid et al. 2006; Arshad et al. 2007; Nadeem et al. 2007, 2009; Chen et al. 2013). Since phytoremediation approach to decontaminate the metal-spiked soils is largely reliant on the profuse growth of roots and the efficient uptake and mobilization of heavy metal ions via prolific root system to different plant parts, stress-induced ethylene at supra-optimal concentration leads to reduced root growth in turn, limiting the proficiency of metal remediating plants (Arshad et al. 2007; Gamalero and Glick 2012).

To counter this physiological crisis, an enzyme ACC deaminase (EC 4.1.99.4) produced by many soil microflora including PSB (Kumar et al. 2009; He et al. 2010), degrades ACC (an immediate precursor for ethylene in plants) into 2-oxobutanoate and ammonia hence decreases the ethylene biosynthesis in plant tissues (Saleem et al. 2007; Shaharoona et al. 2007; Zahir et al. 2009). Ammonia released in this way is utilized by ACC deaminase-expressing organisms as nitrogen source for growth (Glick 2005). In addition, while attached with the plant roots, ACC deaminase-containing bacteria act as a sink for ACC ensuring that ethylene level may not increased to the point where root growth and development is impaired (Glick 1995). Thus, bacterial ACC deaminase-induced extensive root proliferation in metal remediating (hyperaccumulator) plants results into efficient phytoremediation processes in metal-polluted soils (Arshad et al. 2007). Several species of ACC deaminase-containing PSB have been isolated and successfully improved the plant growth under metal stress (Ganesan 2008; Jiang et al. 2008; Sun et al. 2009).

## Exploiting PSB in phytoremediation of metal-stressed soils

 In various studies, growth promoting effects of PSB are well established both in unpolluted and polluted soils when used as inoculants (Ma et al. 2011a, b; Oves et al. 2013). However, degree of their impact on different plants varies depending upon plant species, bacterial species, soil types and environmental factors. In metalliferous soils, several authors have studied phytoremediation using PSB as bioinoculants to remove different heavy metals from soils. Worldwide, the research in this direction is currently being carried out considering various aspects to overcome hurdles which impede the efficient removal of metal contaminants. In Table 3, various phytoremediation studies have been listed to show effects of different PSB using different plant species and metals. Many insights can be drawn following analyses of these studies:

**Table 3:** Phosphate-solubilizing bacteria (PSB) mediated metal remediation and plant growth promotion

PSB	Plant	Heavy metals	Conditions	Role of PSB (Mode of metal remediation)	References
Pseudomonas aeruginosa strain OSG41	Chickpea (*Cicerarietinum*)	Cr	Pots	Increased the dry matter, symbiotic traits, grain yield and grain protein of chickpea plants in the presence of chromium and decreased the uptake of chromium by 36, 38 and 40 % in roots, shoots and grains, respectively (Phytostabilization)	Oves et al. (2013)
*Acinetobacter haemolyticus* RP19	Pearl millet (*Pennisetum glaucum*)	Zn	Pots	Increased significantly root length, shoot length, fresh weight and root biomass (Phytostabilization)	Misra et al. (2012)
*Pseudomonas* sp. A3R3	*Alyssumserpyllifolium*, *Brassicajuncea*	Ni	Pots	Increased significantly the biomass (*B. juncea*) and Ni content (*A. serpyllifolium*) in plants grown in Ni-stressed soil (Phytoextraction)	Ma et al. (2011a)
*Pseudomonas* sp. TLC 6-6.5-4	*Zeamays*, *Helianthusannuus*	Cu	Pots	Significantly increased copper uptake by plants and also enhanced the biomass of maize (Phytoextraction)	Li and Ramakrishna (2011)
*Psychrobacter* sp. SRS8	*Ricinuscommunis*, *Helianthusannuus*	Ni	Pots	Stimulated plant growth and Ni accumulation in both plant species with increased plant biomass, chlorophyll and protein content (Phytoextraction)	Ma et al. (2011b)
*Arthrobacter* sp. MT16, *Microbacterium* sp. JYC17, *Pseudomonaschlororaphis* SZY6, *Azotobactervinelandii* GZC24, *Microbacteriumlactium* YJ7	*Brassica napus*	Cu	Gnotobiotic condition	Increased (16–41 %) root length (Phytoextraction)	He et al. (2010)
*Bacillus* species PSB10	Chickpea (*Cicerarietinum*)	Cr	Pots	Significantly improved growth, nodulation, chlorophyll, leghemoglobin, seed yield and grain protein and reduced the uptake of chromium in roots, shoots and grains (Phytostabilization)	Wani and Khan (2010)
*Pseudomonas* sp. SRI2, *Psychrobacter* sp. SRS8, *Bacillus* sp. SN9	*Brassicajuncea*, *Brassicaoxyrrhina*	Ni	Pots	Increased the biomass of the test plants and enhanced Ni accumulation in plant tissues (Phytoextraction)	Ma et al. (2009a)
*Psychrobacter* sp. SRA1, *Bacilluscereus* SRA10	*Brassicajuncea*, *Brassicaoxyrrhina*	Ni	Pots	Enhanced the metal accumulation in plant tissues by facilitating the release of Ni from the non-soluble phases in the soil (Phytoextraction)	Ma et al. (2009b)
Achromobacter xylosoxidans strain Ax10	Brassica juncea	Cu	Pots	Significantly improved Cu uptake by plants and increased the root length, shoot length, fresh weight and dry weight of plants (Phytoextraction)	Ma et al. (2009c)
*Pseudomonas* sp.	Chickpea	Ni	Pots	Enhanced fresh and dry weight of plants even at 2 mM nickel concentration (Phytostabilization)	Tank and Saraf (2009)
*Enterobacteraerogenes* NBRI K24, *Rahnellaaquatilis* NBRI K3	*Brassica* *juncea*	Ni, Cr	Pots	Increased plant root length, dry weight, leaf protein and chlorophyll content with Ni and Cr uptake (Phytostabilization)	Kumar et al. (2009)
*Bacillusweihenstephanensis* strain SM3	*Helianthus* *annuus*	Ni, Cu, Zn	Pots	Increased plant biomass and the accumulation of Cu and Zn in the root and shoot systems, also augmented the concentrations of water soluble Ni, Cu and Zn in soil with their metal mobilizing potential (Phytoextraction)	Rajkumar et al. (2008)
*Pseudomonasaeruginosa* strain MKRh3	Black gram	Cd	Pots	Plants showed lessened cadmium accumulation, extensive rooting, and enhanced plant growth (Phytostabilization)	Ganesan (2008)
*Enterobacter* sp. NBRI K28, mutant NBRI K28 SD1	*Brassica* *juncea*	Ni, Cr, Zn	Pots	Improved plant growth parameters such as biomass, chlorophyll and protein and increased Ni, Cr and Zn uptake (Phytoextraction)	Kumar et al. (2008)
*Burkholderia* sp. J62	*Lycopersicon esculentum*	Pb, Cd	Pots	Increased root and shoot dry weight as well as Pb and Cd uptake (Phytoextraction)	Jiang et al. (2008)
*Bacillussubtilis* SJ-101	*Brassica juncea*	Ni	Growth chamber	Facilitated Ni accumulation (Phytoextraction)	Zaidi et al. (2006)
*Pseudomonas* sp., *Bacillus* sp.	Mustard	Cr	Pots	Stimulated plant growth and decreased Cr(VI) content (Phytostabilization)	Rajkumar et al. (2006)
*Pseudomonas* *fluorescens*	Soybean	Hg	Greenhouse	Increased plant growth (Phytostabilization)	Gupta et al. (2005)
*Pseudomonas* sp.	Soybean, mungbean, wheat	Ni, Cd, Cr	Pots	Promotes growth of plants (Phytostabilization)	Gupta et al. (2002)

1. Most of the laboratory or green house studies have employed plants of *Brassicaseae* family in conjunction with PSB because plant species of this family (hyperaccumulator plants) have been reported to accumulate substantial amount of metals in their tissues.

2. Diverse species of PSB have been used in these metal phytoremediation studies. However, species like *Pseudomonasaeruginosa*, being an opportunistic human pathogen, poses a challenge to be released in the soil environment (Walker et al. 2004). Hence, ethical and juridical considerations are needed if they are to be used as inoculants in fields.

3. Among environmentally toxic metals, only few metals such as, Ni, Cu, Zn and Cr have been studied extensively while other toxicologically important metals, e.g., As, Cd, Hg and Pb are least considered. Therefore, phytoremediation studies concerning other metals would reveal new challenges, insights and problems leading to pave ways for further research in this course.

4. Both approaches of metal remediation, phytostabilization and phytoextraction have been implicated in these studies. As the plants growing in metal-stressed soils are weakened due to metal-induced physiological damage, they become prone to diseases and pests attack. In practicing phytoextraction strategy, it is paramount important that selected plants must exhibit resistance to phytopathogens in order to smoothly function in metal-stressed soils. Moreover, further exploration and application of PSB strains, possessing additional traits which confer resistance to plants against various diseases, would be a better choice for metal phytoextraction.

## Conclusions

Efficiency of phytoremediation of metal-polluted soils is chiefly determined by the metal bioavailability which in turn increases the metal uptake by plants. Hence, PSB compared with other plant growth promoting bacteria would be marvelous alternatives to boost this process as organic acids, and bio-surfactants secreted by these organisms solubilize sparingly soluble metal complexes, consequently increase bioavailability of metals and nutrient supply to soils. Thus, PSB with multifunctional activities (such as production of siderophore, IAA, ACC deaminase, organic acids and anti-pathogen metabolites) are better choice in assisting the phytoremediation process in metal-contaminated soils.
